# Comprehensive strategy for identifying extracellular vesicle surface proteins as biomarkers for chronic kidney disease

**DOI:** 10.3389/fphys.2024.1328362

**Published:** 2024-02-06

**Authors:** Nahuel Aquiles Garcia, Hernan Gonzalez-King, Maiken Mellergaard, Soumyalekshmi Nair, Carlos Salomon, Aase Handberg

**Affiliations:** ^1^ GECORP, Buenos Aires, Argentina; ^2^ Research and Early Development, Cardiovascular, Renal and Metabolism (CVRM), BioPharmaceuticals R&D, AstraZeneca, Gothenburg, Sweden; ^3^ Department of Clinical Biochemistry, Aalborg University Hospital, Aalborg, Denmark; ^4^ Department of Clinical Medicine, The Faculty of Medicine, Aalborg University, Aalborg, Denmark; ^5^ Translational Extracellular Vesicles in Obstetrics and Gynae-Oncology Group, University of Queensland, Brisbane, QLD, Australia

**Keywords:** extracellular vesicles, chronic kidney disease, obesity, surface proteins, biomarkers

## Abstract

Chronic kidney disease (CKD) poses a significant health burden worldwide. Especially, obesity-induced chronic kidney disease (OCKD) is associated with a lack of accuracy in disease diagnostic methods. The identification of reliable biomarkers for the early diagnosis and monitoring of CKD and OCKD is crucial for improving patient outcomes. Extracellular vesicles (EVs) have emerged as potential biomarkers in the context of CKD. In this review, we focused on the role of EVs as potential biomarkers in CKD and OCKD and developed a comprehensive list of EV membrane proteins that could aid in the diagnosis and monitoring of the disease. To assemble our list, we employed a multi-step strategy. Initially, we conducted a thorough review of the literature on EV protein biomarkers in kidney diseases. Additionally, we explored papers investigating circulating proteins as biomarkers in kidney diseases. To further refine our list, we utilized the EV database Vesiclepedia.org to evaluate the qualifications of each identified protein. Furthermore, we consulted the Human Protein Atlas to assess the localization of these candidates, with a particular focus on membrane proteins. By integrating the information from the reviewed literature, Vesiclepedia.org, and the Human Protein Atlas, we compiled a comprehensive list of potential EV membrane protein biomarkers for CKD and OCKD. Overall, our review underscores the potential of EVs as biomarkers in the field of CKD research, providing a foundation for future studies aimed at improving CKD and OCKD diagnosis and treatment.

## 1 Introduction

Obesity has become a global epidemic and strongly associates with development of various chronic diseases, including chronic kidney disease (CKD) (Carbone et al., 2018). When obesity is related to the development of CKD, it is known as obesity-induced chronic kidney disease (OCKD). OCKD is characterized by the intricate interplay between obesity-related metabolic disturbances and renal dysfunction (Docherty et al., 2020). Excessive adipose tissue results in a state of chronic inflammation and metabolic dysfunction, marked by insulin resistance, dyslipidemia, and altered adipokine secretion. These factors collectively contribute to endothelial dysfunction, oxidative stress, and inflammation within the kidney. The resultant renal damage includes glomerular hypertrophy, hyperfiltration, and impaired autoregulation, ultimately leading to proteinuria and albuminuria (Docherty et al., 2020). Moreover, the adipose tissue itself becomes an endocrine organ, releasing cytokines and adipokines that can directly affect renal function and structure. Adiponectin, leptin, and inflammatory molecules such as TNF-alpha play pivotal roles in modulating renal pathways involved in fibrosis, inflammation, and oxidative stress (Brennan et al., 2021). Recent studies have highlighted the importance of targeting obesity not only as a risk factor for CKD development but also as a potential therapeutic avenue for managing CKD progression. Strategies involving weight loss, lifestyle modifications, and interventions to reduce systemic inflammation could prove pivotal in mitigating the impact of obesity on CKD (Brennan et al., 2021). Thus, the intricate interplay between obesity and CKD underscores the significance of preventive measures and innovative treatments to combat the rising burden of OCKD. The prevalence of OCKD highlights the need for timely and accurate diagnostic methods to facilitate early intervention and improve patient outcomes.

## 2 Kidney disease diagnostic methods

Traditional approaches to diagnostic kidney health like Glomerular filtration rate (GFR) estimation, albuminuria measurement, and serum creatinine levels are widely used but may have reduced accuracy in obese individuals. GFR is a key indicator of kidney function. It reflects the rate at which the kidneys filter waste products and excess fluids from the blood. The most common method to estimate GFR is by calculating it using creatinine-based equations, such as the Cockcroft-Gault equation or the Modification of Diet in Renal Disease (MDRD) equation. GFR estimation equations may be less accurate in obese individuals due to differences in muscle mass, which can affect creatinine levels (Chang et al., 2018). These equations were initially developed and validated in non-obese populations, leading to potential inaccuracies when applied to obese individuals. In obesity, alterations in creatinine production, tubular secretion, and plasma volume can affect the accuracy of GFR estimation.

Albuminuria refers to the presence of excess albumin in the urine and is an early marker of kidney damage. It is commonly measured using a spot urine sample or a 24-hour urine collection. When using spot urine, the albumin-to-creatinine ratio (ACR) is often calculated to standardize the results. Obesity can cause fluctuations in urinary albumin excretion, leading to challenges in accurately assessing kidney damage (Rosenstock et al., 2018). The diagnostic performance of albuminuria may be affected by other factors, such as urinary tract infections, exercise, or dehydration.

Serum creatinine levels are routinely measured in clinical practice as a part of standard health check-ups. Elevated creatinine levels in the blood indicate impaired kidney function. Serum creatinine levels alone may not reliably detect early kidney damage, as the kidneys can maintain normal creatinine levels until significant renal function loss occurs (Gerchman et al., 2009). Creatinine levels can be influenced by factors like muscle mass, diet, and hydration status, which can be altered in obesity.

**FIGURE 1:**
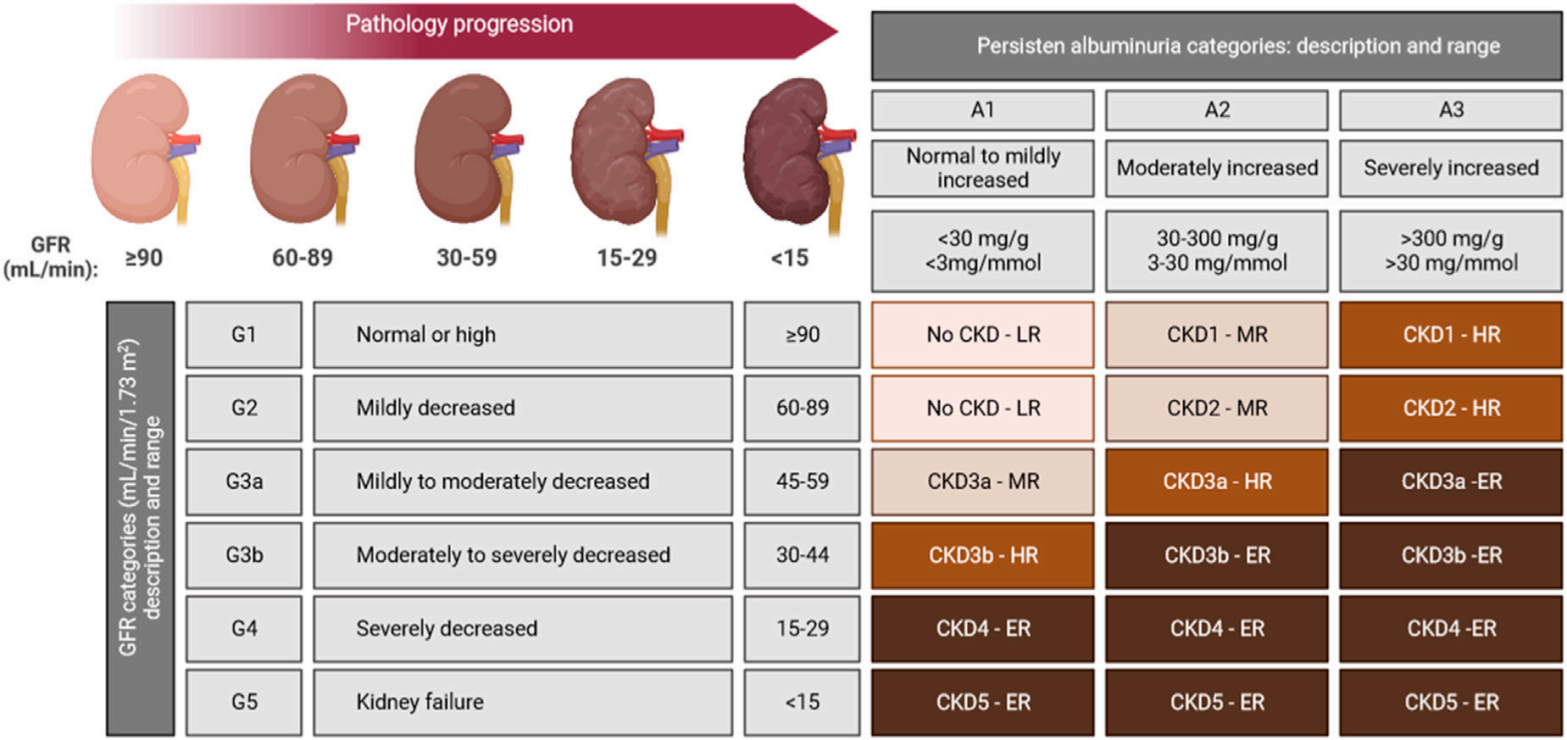
CKD staging system based on both GFR and albuminuria levels are indicated in each cell. Colour-code goes from light brown (LR: low-risk), to dark brown (ER: extreme-risk) through intermediates brown colours (MR: mild-risk; HR: high-risk), ranking the risk for five of some of the most common CKD outcomes: cardiovascular disease (CVD), kidney failure treated by dialysis and transplantation, acute kidney injury, and progression of kidney disease (Stevens et al., 2013).

Based on the mentioned, it is possible to describe a staging system for CKD evaluation and management (Figure 1).

New biomarkers and non-invasive imaging modalities show promise in improving early detection and monitoring, but their clinical utility and cost-effectiveness need further investigation. Novel biomarkers have gained significant interest in the context of OCKD due to their potential to address certain limitations of traditional diagnostic methods. These biomarkers offer additional information beyond the standard markers like creatinine and albuminuria, and they have the potential to improve early detection, risk stratification, and monitoring of OCKD.

Cystatin C is a small protein produced by all nucleated cells, and it is freely filtered by the glomeruli. Unlike creatinine, it is less influenced by muscle mass, making it potentially more reliable in obese individuals. Studies have shown that cystatin C-based estimates of glomerular filtration rate (eGFR) can outperform creatinine-based equations in detecting early kidney dysfunction, especially in individuals with a higher BMI (Kar et al., 2018). Despite being less influenced by muscle mass, cystatin C levels can still be affected by factors like inflammation, thyroid dysfunction, and certain medications, potentially leading to false results. The cost of cystatin C assays may be higher than standard tests, limiting its routine use in some healthcare settings.

Neutrophil Gelatinase-Associated Lipocalin (NGAL) is a small protein involved in the response to kidney injury and inflammation. It can be detected in the urine or blood, early after kidney damage, making it a potential early marker of kidney injury and CKD. Studies have shown that NGAL levels are elevated in obese individuals with CKD, suggesting its relevance in OCKD diagnosis and monitoring (Sen et al., 2021). Nevertheless, NGAL levels can also be increased in response to non-renal conditions, such as infections or inflammatory processes, which may limit its specificity in diagnosing OCKD (Sen et al., 2021). The use of NGAL as a standalone biomarker may not be sufficient for diagnosing OCKD, and likely need to be combined with other markers for better accuracy.

Kidney injury molecule 1 (KIM-1) is a transmembrane protein expressed in the kidney tubules. It is highly upregulated in response to kidney injury and can be detected in urine early after damage occurs, making it a potential marker for early detection of kidney injury in obese individuals. However, like NGAL, KIM-1 can be elevated in response to non-renal conditions, affecting its specificity in diagnosing OCKD (Polidori et al., 2020). Other novel biomarkers, such as interleukin-18 (IL-18), and fatty acid-binding protein (FABP), have been explored for their potential in detecting early kidney damage in obesity (Xu et al., 2015; Hirooka and Nozaki, 2021). These biomarkers are also related to different aspects of kidney injury and repair processes. Novel biomarkers show promise in addressing some of the limitations of traditional methods in diagnosing OCKD.

However, it is essential to recognize that no single biomarker is sufficient for a comprehensive assessment of kidney function and injury.

Imaging techniques, such as renal ultrasound, magnetic resonance imaging (MRI), or computed tomography (CT) scans, can provide valuable information about kidney structure and help identify structural abnormalities or kidney stones. Imaging modalities are more useful for identifying anatomical abnormalities rather than assessing kidney function directly (van Niel et al., 2022). While non-invasive, these imaging techniques may not be sensitive enough to detect early kidney damage.

## 3 EVs in kidney disease

**FIGURE 2:**
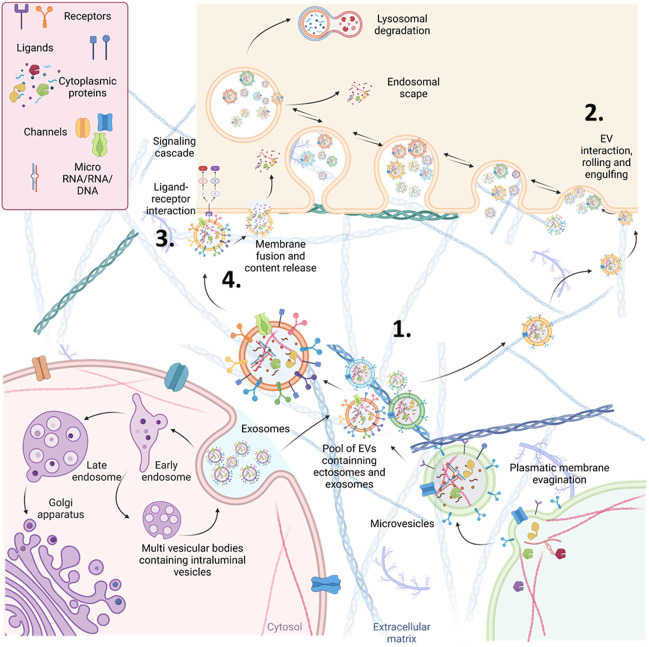
Schematic representation of the different origins and action mechanisms for EVs. 1. EVs can be produced from the early endosomal pathway (called small EVs or exosomes around 30–130 nm of diameter) or by evaginations of the plasmatic membrane (EVs called microvesicles or ectosomes around 130–1,000 nm of diameter). 2. EVs can be engulfed by other cells through pinocytosis or phagocytosis. After being engulfed, EVs, now inside an endosome, can fuse with the membrane of the endosome and release their content (endosomal scape) degraded by fusion with lysosomes or placed again in the extracellular space (recycling). 3. EVs can also interact directly with receptors to trigger signalling cascades in the receiving cells. 4. Alternatively, they can directly fuse with the plasmatic membrane of the receiving cells, releasing their cytosolic cargo.

EVs constitute a diverse group of vesicles that exhibit variations in size, content, and functionality (van Niel et al., 2022). They are categorized according to their biogenesis and dimensions into three primary classes: small EVs, commonly known as exosomes (30–150 nm), microvesicles, also referred to as ectosomes (150–1,000 nm), and apoptotic bodies (>1,000 nm), as depicted in Figure 2. A wide range of cell types, including renal cells, release these EVs, and they can be detected in various bodily fluids, such as blood (referred to as circulating EVs or cEVs) and urine (referred to as urine EVs or uEVs). EVs have gained significant recognition as crucial mediators of intercellular communication since they transport an array of biomolecules capable of targeted delivery to recipient cells (van Niel et al., 2022).

**FIGURE 3:**
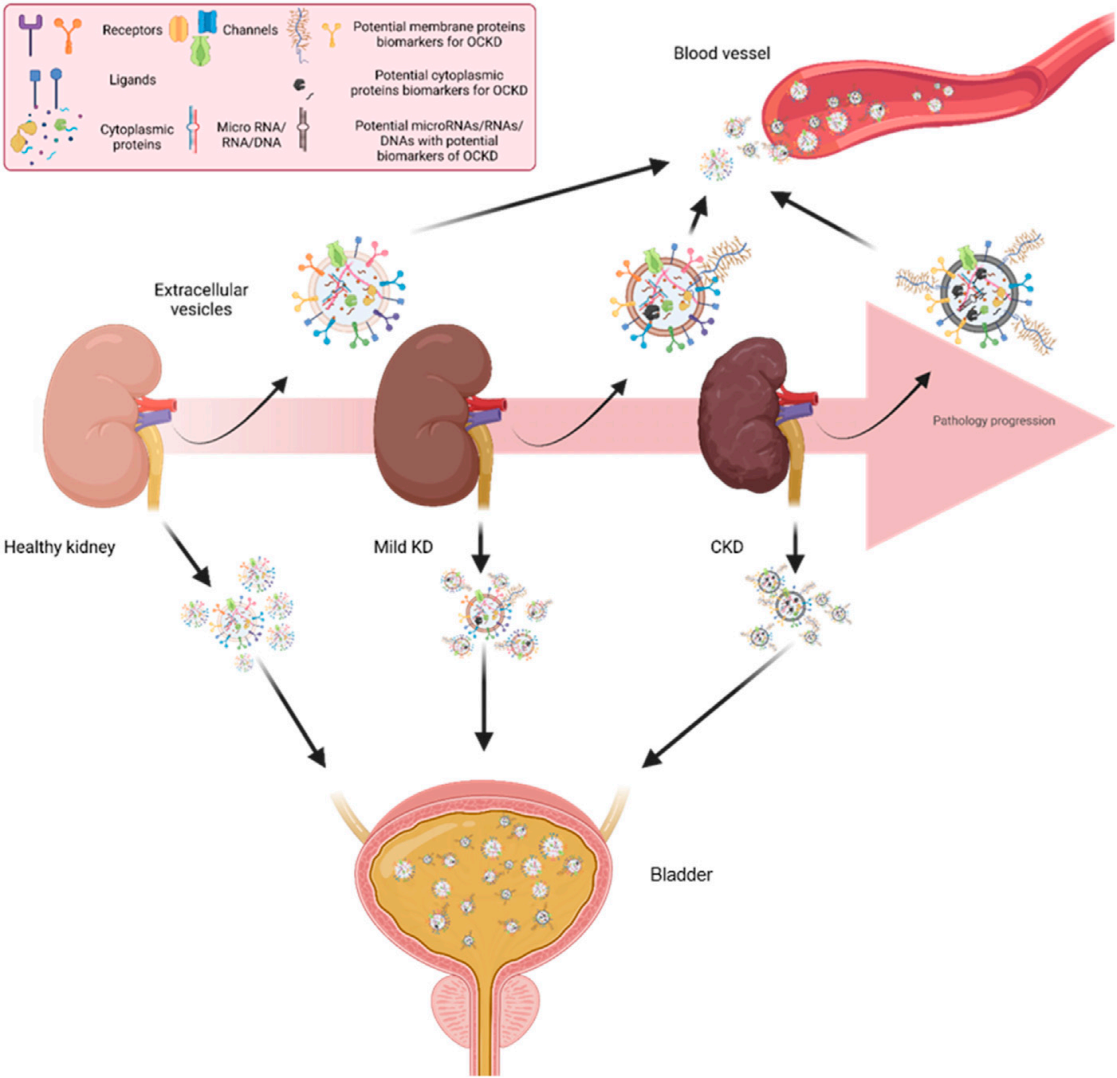
Kidney cells releasing EVs to the circulation and urine. Across different stages of CKD, EVs released from kidney cells to urine and circulation could have different patterns of surface proteins. This differential expression of EV surface proteins could be used as CKD biomarkers by measuring patients’ blood and urine samples. KD: Kidney disease. CKD: Chronic kidney disease.

The role of EVs in CKD and OCKD has been widely investigated, and some studies have suggested that EVs can be used as biomarkers for the diagnosis and monitoring of OCKD (Shibata, 2021; Yin et al., 2023). The regulation of EV synthesis and secretion in various stages of kidney disease could be linked to cellular stress, inflammation, and fibrotic responses characteristic of each disease stage. Thus, the cargo of EVs secreted by kidney cells can reflect the kidney’s pathophysiology. cEVs and uEVs in CKD and OCKD patients has been shown to carry specific proteins that are related to lipid metabolism, oxidative stress, inflammation, and fibrosis, which are hallmarks of pathology progression (D et al., 2023; Grange and Bussolati, 2022) (Figure 3). uEVs and cEVs have been investigated as potential diagnostic tools in CKD. Both urinary and circulating EVs have their strengths and limitations as potential biomarkers (Table 1). Combining information from both types of EVs might provide a more comprehensive understanding of OCKD and its systemic effects on the body. However, more research is needed to fully elucidate and validate the diagnostic and prognostic potential of these biomarkers for OCKD and other diseases (Blijdorp et al., 2022). In this regard, the International Society for Extracellular Vesicles has presented a position paper related to uEVs (Erdbrugger et al., 2021). Numerous research studies have been dedicated to the exploration of biomolecules with diagnostic significance within EVs originating from patients with CKD and OCKD. These works primarily use conventional techniques for EVs isolation and purification, such as ultracentrifugation, followed by comprehensive omics analyses (e.g., proteomics, RNAseq, etc.). These methods are indispensable for identifying potential biomolecular candidates; however, their intricate nature renders them less suitable for clinical translation.

**TABLE 1:** uEVs vs. cEVs.

Urinary EVs		Circulating EVs
Strong points	• Proximity to Kidney Tissue: uEVs are directly derived from renal cells and reflect the local microenvironment, providing specific information about the kidney’s health and function	• Systemic Insights: cEVs can carry cargo from various organs, providing a systemic perspective of CKD pathogenesis and associated comorbidities
	• Non-invasive Sample Collection: Obtaining urine samples is relatively non-invasive, making uEVs a convenient biomarker source for disease monitoring	• Stable in Circulation: cEVs are protected from enzymatic degradation in circulation, making them a robust source of stable biomarkers
	• Enriched Biomarker Pool: uEVs carry a rich cargo of renal-specific proteins, miRNAs, and other molecules, providing potential disease-specific biomarkers	
Weak points	• Variable Concentrations: uEVs’ concentration can vary due to factors like hydration status, urinary flow rate, and sample processing methods, potentially affecting their reliability	• Dilution Effect: cEVs from the kidneys might be diluted in the bloodstream by EVs from other organs, reducing the specificity for renal-specific events
	• Limited Systemic Information: uEVs primarily represent kidney-specific events and may not capture the broader systemic aspects of CKD	• Difficult Sample Collection: Obtaining blood samples can be more invasive and challenging for certain patient populations, potentially limiting its widespread clinical application
	• Potential Contamination: There is a possibility of contamination from the lower urinary tract, affecting the specificity of uEVs as a CKD biomarker	

In this context, emerging technologies like EV-Array (Baek and Jorgensen, 2017) and Exoview offer streamlined and efficient approaches for the study of EV surface proteins (Breitwieser et al., 2022). EV-Array, grounded in protein microarray technology, enables the high-throughput detection and phenotyping of small EVs, even from unpurified starting materials. Exoview, on the other hand, introduces an innovative method for characterizing EV surface proteins by combining nanoparticle tracking analysis (NTA) to determine size and concentration with a microarray-based assay for surface protein analysis. The microarray-based assay employs immobilized antibodies on a glass slide to capture specific EV proteins, which are subsequently detected using fluorescently labeled secondary antibodies. The resulting protein profiles of EVs yield valuable insights into their underlying biology (Breitwieser et al., 2022). Advancements in flow cytometry have given rise to high-sensitive flow cytometry (HS-FCM), offering superior sensitivity and specificity compared to traditional flow cytometry methods (Botha et al., 2021). HS-FCM achieves this through the use of smaller sample volumes and specialized instrumentation, minimizing background noise and enhancing signal detection. Furthermore, HS-FCM excels in providing information on multiple surface proteins concurrently (Nielsen et al., 2019). It can directly identify and quantify the expression of numerous proteins on individual EVs in plasma, without the need for preceding purification steps, thereby enabling a more comprehensive analysis of EV populations.

In a recent study (Garcia et al., 2023), we introduced a method to identify EV surface proteins with the potential to serve as biomarkers in non-alcoholic fatty liver disease (NAFLD). Employing a similar methodology, this review aims to compile a list of prospective EV surface protein biomarkers that could facilitate the diagnosis and monitoring of CKD and OCKD. This review presents a comprehensive summary of the current knowledge regarding EV surface proteins as potential biomarkers for kidney diseases. To accomplish this, we undertook a multifaceted approach: (I) We conducted a thorough review of the scientific literature exploring EV proteins as biomarkers in kidney pathologies, along with research papers investigating circulating proteins as biomarkers in CKD and OCKD, as well as papers and reviews summarizing the principal proteins involved in CKD and OCKD biology. (II) To identify potential candidates, we employed the EV database Vesiclepedia.org, qualifying each protein and selecting those that had previously been found in EVs from various cell and tissue sources. (III) Finally, we consulted the Human Protein Atlas (https://www.proteinatlas.org/) to ascertain the localization of each protein, with a focus on membrane proteins that can be easily detected using the aforementioned technologies (EV-Array, Exoview, and HS-FCM). The resulting list was then categorized based on biological function and technical applications related to these EV proteins. The outcome of this comprehensive process is presented in Table 2, which showcases 60 protein candidates that could potentially serve as EV surface biomarkers in CKD and OCKD. Table 2 features both surface proteins that have been directly confirmed in EVs derived from key tissues in CKD and OCKD, and those that are anticipated to be found in EVs from these crucial tissues (Table 2, column 4). In the next section, we show a short description of each candidate, here the reader will note that some targets have been found in uEVs and others in cEVs. Thus, to clarify, as mentioned before, in a research stage investigating the biomarker potential of these EV proteins in CKD and OCKD, it would be convenient to assay these candidates in both uEVs and cEVs preparations. Classiclal methods to diagnose kidney disease present more biases when applied to obese patients. Nevertheless, the existence of a complex interplay between CKD and OCKD, make it difficult to discriminate potential biomarkers that separately produce information among both conditions. Thus, the listed proteins identified by our method could be used as potential biomarkers for CKD and/or OCKD.

**TABLE 2:** EV protein candidates related to kidney disease.

Mechanism	Name	Description	Direct corroboration	References
early detection biomarker	HAVCR1	Mediates fatty acids uptake by renal tubular cells	yes	Han et al. (2002), Khreba et al. (2019), Chen et al. (2023)
	WNT4	kidney tubular injury after ischemia/reperfusion	no	Zhang et al. (2015), Zhao et al. (2016)
	LCN2	kidney tubular injury biomarker (FDA approved)	yes	Devarajan (2007), Ugarte et al. (2021), Yang et al. (2022)
	TNFR1	circulating biomarker for kidney inflammation	yes	Hawari et al. (2004), Zabetian and Coca (2021)
	TNFR2	potential biomarker for diabetic kidney disease	no	Murakoshi et al. (2020)
	EGF	urine levels related with CKD progression	no	Isaka (2016), Frawley and Piskareva (2020)
	EGFR	early fibrosis biomarker in CKD	no	Frawley and Piskareva (2020), Harris (2021)
Biomarker	NKCC2	potential biomarker for kidney injury	yes	Riazi et al. (2006), Raimondo et al. (2020), Wu et al. (2021)
	ACE	proposed biomarker for kidney disease	yes	Mizuiri and Ohashi (2015), Tong et al. (2018)
	AGTR1	found in EVs derived from mesangial cells	yes	da Silva Novaes et al. (2019), Pironti et al. (2015)
	AGTR2	found in EVs derived from mesangial cells	yes	da Silva Novaes et al. (2019), Pironti et al. (2015)
	AHSG	proposed as uEVs marker for acute kidney injury	yes	Zhou et al. (2006), Magalhaes et al. (2021), Chekol Abebe et al. (2022)
	SLC26A4	uEVs levels related to hypertensive disorders	yes	Wall et al. (2020), Ochiai-Homma et al. (2021)
	SLC9A3	marker of tubule injury in acute renal failure	no	du Cheyron et al. (2003), Barros and Carvajal (2017)
	SLC12A1	uEVs levels as biomarker for Gitelman and Bartter syndromes	yes	Corbetta et al. (2015)
	SLC12A3	uEVs has been proposed as biomarkers for primary aldosteronism	yes	van der Lubbe et al. (2012)
	SCNN1	uEVs levels regulated by mineralocorticoid administration	yes	Ochiai-Homma et al. (2021)
	NPHS1	found in uEVs in patients with Renal Injury	yes	Kandasamy et al. (2014), Gilani et al. (2017)
	podocin	found in uEVs in patients with Renal Injury	yes	Gilani et al. (2017), Pereira et al. (2019), Musante et al. (2020)
	MUC1	proposed biomarker for renal dysfunction	yes	Pisitkun et al. (2004), Zhang et al. (2017)
	AQP2	uEVs levels related to leishmaniasis	yes	Sonoda et al. (2009)
	AQP1	uEVs levels related to renal ischemia-reperfusion injury	yes	Oliveira et al. (2011), Oshikawa et al. (2016)
	PON1	proposed as biomarker for CKD progression	yes	Deakin et al. (2002), Taler-Vercic et al. (2020), Du et al. (2023), Watanabe et al. (2023)
	PLAUR	kidney inflammation indicator	yes	Hayek et al. (2020), Porcelli et al. (2021), Sudhini et al. (2022)
	UMOD	uEVs levels related to general kidney function	yes	Pisitkun et al. (2004), Steubl et al. (2016)
metabolism	SGLT2	glomerular hypertension and hyperfiltration	no	Docherty et al. (2020)
	FABP3	urinary levels related to kidney injury	no	Dihazi et al. (2016), Yu et al. (2022)
	TRPC6	uEVs levels associated to glomerular disease	yes	Hogan et al. (2014), Staruschenko et al. (2019)
	LRP2	mediated tubuloglomerular alterations in high-fat diet	yes	Pisitkun et al. (2004), Cabezas et al. (2011), Kuwahara et al. (2016)
	CUBN	mediates the ectopic fat accumulation in the kidney	yes	Hogan et al. (2014), Lubojemska et al. (2021)
	GLUT1	related to the development of diabetic kidney disease	no	Gnudi and Raij (2006), Wasik and Lehtonen (2018)
	GLUT2	central regulator in the pathogenesis of diabetic kidney disease	no	Ahmad et al. (2022)
	GLUT4	related to diabetic kidney disease	no	Garcia et al. (2016), Piwkowska et al. (2022)
	TM6SF2	Associated with higher eGFR in obese children	no	Marzuillo et al. (2020), Liu et al. (2023)
	CNR1	contribute to both diabetic and non-diabetic CKD	no	Arceri et al. (2023)
Adipose tissue crosstalk	Adiponectin	proposed biomarker of renal dysfunction	yes	Phoonsawat et al. (2014), Gustafson et al. (2015), Przybycinski et al. (2020), Song et al. (2020)
	HSD11B2	effects on the kidney and vasculature	no	Bailey (2017)
	CD36	Related to fat accumulation in kidney	yes	Yang et al. (2017), Botha et al. (2018), Garcia et al. (2019)
	PTHrP	Injured kidney increase PTHrP in blood	no	Ki et al. (2016), Hu et al. (2021)
	PTH1R	mediates cachexia in AT in models of kidney failure	no	Ki et al. (2016), Hu et al. (2021)
	PHGDH	regulates metabolic disorders in the kidney and liver in obesity	no	Chen et al. (2022)
	Perilipin A	Impacts Acute Kidney Injury via Regulation of PPARα	yes	Eguchi et al. (2016), Xu et al. (2021)
fibrosis	PROM1	chronic glomerular damage	yes	Dimuccio et al. (2020), Hu et al. (2022)
	KLOTHO	related with kidney fibrosis	yes	Zou et al. (2018), Grange et al. (2020)
	NOTCH1	involved in CKD fibrosis	no	Sassoli et al. (2011), Olsauskas-Kuprys et al. (2013), Gonzalez-King et al. (2017), Han et al. (2017), Gonzalez-King et al. (2022), Zhou et al. (2022)
	NOTCH2	involved in CKD fibrosis	no	Sassoli et al. (2011), Olsauskas-Kuprys et al. (2013), Gonzalez-King et al. (2017), Han et al. (2017), Gonzalez-King et al. (2022), Zhou et al. (2022)
	DLL1	involved in CKD fibrosis	no	Sassoli et al. (2011), Olsauskas-Kuprys et al. (2013), Gonzalez-King et al. (2017), Han et al. (2017), Gonzalez-King et al. (2022), Zhou et al. (2022)
	DLL3	involved in CKD fibrosis	no	Sassoli et al. (2011), Olsauskas-Kuprys et al. (2013), Gonzalez-King et al. (2017), Han et al. (2017), Gonzalez-King et al. (2022), Zhou et al. (2022)
	DLL4	involved in CKD fibrosis	no	Sassoli et al. (2011), Olsauskas-Kuprys et al. (2013), Gonzalez-King et al. (2017), Han et al. (2017), Gonzalez-King et al. (2022), Zhou et al. (2022)
	JAG1	involved in CKD fibrosis	no	Sassoli et al. (2011), Olsauskas-Kuprys et al. (2013), Gonzalez-King et al. (2017), Han et al. (2017), Gonzalez-King et al. (2022), Zhou et al. (2022)
	JAG2	involved in CKD fibrosis	no	Sassoli et al. (2011), Olsauskas-Kuprys et al. (2013), Gonzalez-King et al. (2017), Han et al. (2017), Gonzalez-King et al. (2022), Zhou et al. (2022)
	TGFB1	involved in CKD fibrosis	no	Sureshbabu et al. (2016), Shelke et al. (2019)
	WNT5A	tubular inflammation in diabetic nephropathy	no	Feng et al. (2018), Martin-Medina et al. (2018), Li et al. (2021a)
	Ctnnb1	involved in CKD fibrosis	no	Li et al. (2021b)
	CXCR4	involved in CKD fibrosis	no	Yuan et al. (2015), Li et al. (2018)
inflammation/immunity	TLR2	induces endothelial dysfunction in CKD patients	no	Speer et al. (2013)
	CR1	related to metabolic associated CKD	yes	Prunotto et al. (2013), Xu et al. (2022)
	WNT1	regulates kidney inflammation through the NF-κB pathway	no	Gross and Zelarayan (2018), Wang et al. (2022b)
	CXCR7	capillary tuft development in kidney	no	Haege et al. (2012)
	CXCL10	potential biomarker for CKD	no	Gao et al. (2020), Gao et al. (2022)

## 4 Early detection biomarkers

In this section we summarized proteins that has been related to the detection of CKD and OCKD in early stages of the pathology progression.

KIM1. Hepatitis A virus cellular receptor 1 (HAVCR1, also known as Kidney injured molecule 1 - KIM1) is a phosphatidylserine receptor that plays an important functional role in regulatory B-cells homeostasis including generation, expansion and suppressor functions. It is highly expressed in renal tubular cells. KIM1 has been proposed as a novel urine biomarker for CKD (Han et al., 2002; Khreba et al., 2019). Recently, Jun et al., showed that KIM1 is necessary for EVs uptake by tubular epithelial cells (Chen et al., 2023).

WNT4. Wnt family member 4 (WNT4) is a ligand for members of the frizzled family of seven transmembrane receptors. It has been proposed as a novel biomarker for the early detection of kidney tubular injury after ischemia/reperfusion in mice (Zhao et al., 2016). WNT4 has been previously reported in EVs derived from human umbilical cord mesenchymal stem cells (Zhang et al., 2015).

LCN2. Lipocalin 2 (LCN2, also known as Neutrophil gelatinase associated lipocalin - NGAL) is a highly conserved secreted adipokine acting as a serum transport protein for small hydrophobic molecules such as fatty acids and steroids. LCN2 is secreted in high amounts into the urine and blood from tubular cells during acute kidney injury (AKI) before serum creatinine rises (Devarajan, 2007). Moreover, Ugarte et al. (2021), showed that LCN2 levels in uEVs could be used in the early detection of tubular injury in type 1diabetes mellitus patients (T1DM). While LCN2 is not a transmembrane protein, we included it in our list because of previous evidence suggesting the possibility to localize LCN2 on membranes surface (Yang et al., 2022).

TNFR1. TNF receptor superfamily member 1A (TNFR1) is a receptor for TNFSF2/TNF-alpha and homotrimeric TNFSF1/lymphotoxin-alpha. It has been proposed as a circulatory biomarker to detect early kidney inflammation in CKD patients (Zabetian and Coca, 2021). Interestingly, TNFR1 has been found in cEVs in human plasma (Hawari et al., 2004).

TNFR2. TNF receptor superfamily member 1B (TNFR2) is a receptor with high affinity for TNFSF2/TNF-alpha and approximately 5-fold lower affinity for homotrimeric TNFSF1/lymphotoxin-alpha. It could be a potential biomarker for the progression of diabetic kidney disease (Murakoshi et al., 2020).

EGF. Epidermal growth factor (EGF) is a potent mitogenic factor that plays an important role in the growth, proliferation and differentiation of numerous cell types. Urinary levels of EGF have been related to CKD progression (Isaka, 2016). Moreover, the role of EVs in EGF signal spread out was corroborated by different groups (Frawley and Piskareva, 2020).

EGFR. Epidermal growth factor receptor (EGFR) is a tyrosine kinase receptor which bind ligands of the EGF family. It could act as an early fibrosis biomarker in CKD (Harris, 2021). Moreover, the role of EVs in EGF signal spread out was corroborated by different groups (Frawley and Piskareva, 2020).

## 5 Already described EV surface proteins as biomarkers

In this section we listed the EV surface proteins that has been described as potential biomarkers in CKD and OCKD.

NKCC2. Solute carrier family 12 member 1 (NKCC2) is a renal sodium, potassium, and chloride ion co-transporter that mediates the transepithelial NaCl reabsorption in the thick ascending limb and plays an essential role in the urinary concentration and volume regulation. NKCC2 levels are altered in OCKD (Riazi et al., 2006; Wu et al., 2021). Raimondo et al., proposed NKCC2 levels in uEVs as potential biomarker for kidney injury Raimondo et al. (2020).

ACE. Angiotensin I converting enzyme (ACE) is a dipeptidyl carboxypeptidase that removes dipeptides from the C-terminus of a variety of circulating hormones, such as angiotensin I, bradykinin or enkephalins, thereby playing a key role in the regulation of blood pressure, electrolyte homeostasis or synaptic plasticity. ACE plasmatic levels has been proposed as biomarkers for kidney disease (Mizuiri and Ohashi, 2015). Interestingly, EVs-mediated transfer of ACE was showed in hypertensive rats to promote vascular smooth muscle cell migration (Tong et al., 2018).

AGTR1 and AGTR2. Angiotensin II receptor type 1 and 2 (AGTR1 and AGTR2) are the receptors for angiotensin II, a vasoconstricting peptide, which acts as a key regulator of blood pressure and sodium retention by the kidney. Both receptors where found on EVs derived from mesangial cell under high glucose treatment (da Silva Novaes et al., 2019). Moreover, cEVs induced by cardiac pressure overload contain functional angiotensin II type 1 receptors (Pironti et al., 2015).

AHSG. Alpha 2-HS glycoprotein (AHSG, also known as Fetuin-A) is a negatively-charged serum glycoprotein which promotes endocytosis, possesses opsonic properties and influences the mineral phase of bone. Urinary fetuin-A peptides were postulated as biomarkers for impaired kidney function in patients with type 2 diabetes (Magalhaes et al., 2021). Additionally, EV AHSG levels in urine could be used as biomarker for detecting acute kidney injury (Zhou et al., 2006). While AHSG is not a transmembrane protein, we still believe it is worth it to include it in our list since there is evidence showed AHSG directly binding the plasmatic membrane surface (Chekol Abebe et al., 2022).

SLC26A4. Solute carrier family 26 member 4 (SLC26A4, also known as Pendrin) is a sodium-independent transporter of chloride and iodide. Pendrin is stimulated by angiotensin II and aldosterone administration via the angiotensin type 1a and the mineralocorticoid receptors, respectively. It is also stimulated in models of metabolic alkalosis (Wall et al., 2020). Pendrin analysis in human uEVs can be useful to understand the pathophysiology of hypertensive disorders (Ochiai-Homma et al., 2021).

SLC9A3. Solute carrier family 9 member A3 (SLC9A3, also known as NHE3) is an epithelial brush border Na/H exchanger that uses an inward sodium ion gradient to expel acids from the cell. NHE3 urinary levels could be used as marker of tubule injury in acute renal failure (du Cheyron et al., 2003). NHE3 could be present in uEVs (Barros and Carvajal, 2017).

SLC12A1. Solute carrier family 12 member 1 (SLC12A1, also known as NKCC2), is a Renal sodium, potassium and chloride ion cotransporter that mediates the transepithelial NaCl reabsorption in the thick ascending limb and plays an essential role in the urinary concentration and volume regulation. uEVs NKCC2 levels has been proposed as biomarker for Gitelman and Bartter syndromes (Corbetta et al., 2015).

SLC12A3. Solute carrier family 12 member 3 (SLC12A3, also known as NCC) an electroneutral sodium and chloride ion cotransporter. In kidney distal convoluted tubules, key mediator of sodium and chloride reabsorption. Levels of NCC in uEVs have been proposed as biomarkers for primary aldosteronism (van der Lubbe et al., 2012).

SCNN1. Sodium channel epithelial 1 subunit alpha (SCNN1) is a sodium permeable non-voltage-sensitive ion channel inhibited by the diuretic amiloride. Mediates the electrodiffusion of the luminal sodium (and water, which follows osmotically) through the apical membrane of epithelial cells. Previous studies have documented acute changes in the ENaC levels in uEVs after mineralocorticoid administration (Ochiai-Homma et al., 2021).

NPHS1. Nephrin adhesion molecule (NPHS1) is a cell adhesion molecule that functions in the glomerular filtration barrier in the kidney. It could be used as biomarker of early glomerular injury (Kandasamy et al., 2014). Nephrin is present in uEVs and has been described as a possible biomarker for renal injury in preeclampsia (Gilani et al., 2017).

NPHS2. Stomatin family member, podocin (NPHS2) plays a role in the regulation of glomerular permeability. Podocin has been proposed as biomarker in cases of focal and segmental glomerulosclerosis (Pereira et al., 2019). Podocin is present in uEVs (Musante et al., 2020) and has been described as a possible biomarker for renal injury in preeclampsia (Gilani et al., 2017).

MUC1. Mucin 1 (MUC1) is a membrane-bound protein, member of the mucin family. Mucins are O-glycosylated proteins that play an essential role in forming protective mucous barriers on epithelial surfaces. In urine, it is a novel biomarker associated with renal dysfunction in the general population (Zhang et al., 2017). uEVs carry MUC1 their on surface (Pisitkun et al., 2004).

AQP1. Aquaporin 1 (AQP1) forms a water-specific channel that provides the plasma membranes of red cells and kidney proximal tubules with high permeability to water. uEVs bring AQP1 on surface (Pisitkun et al., 2004) and its levels are related to renal ischemia-reperfusion injury (Sonoda et al., 2009).

AQP2. Aquaporine 2 (AQP2) forms a water-specific channel that provides the plasma membranes of red cells and kidney proximal tubules with high permeability to water. AQP2 in urine is reportedly a good marker for the effect of vasopressin on the renal collecting ducts, and many studies have measured the level of AQP2 in whole urine of patients with various diseases related to imbalance of water homeostasis (Oshikawa et al., 2016). Oliveira et al. have shown that a reduction in the level of AQP2 in uEVs can be a useful biomarker of urinary concentrating defects in patients with American cutaneous leishmaniasis (Oliveira et al., 2011).

PON1. Paraoxonase 1 (PON1) is a member of the paraoxonase family of enzymes and exhibits lactonase and ester hydrolase activity. Plasmatic activity of PON1 is a biomarker for the progression of CKD (Watanabe et al., 2023). PON1 has been reported in cEVs (Du et al., 2023). PON1 is not a transmembrane protein, but strong evidence suggest that it can be find in the plasmatic membrane surface (Deakin et al., 2002; Taler-Vercic et al., 2020).

PLAUR. Plasminogen activator urokinase receptor (PLAUR) acts as a receptor for urokinase plasminogen activator. Circulating levels of PLAUR can be indicative of kidney inflammation and acute kidney injury (Hayek et al., 2020; Sudhini et al., 2022). PLAUR levels in cEVs have been proposed as biomarker for metastatic melanoma patients (Porcelli et al., 2021).

UMOD. Uromodulin (UMOD) is the most abundant protein in mammalian urine under physiological conditions. Its excretion in urine follows proteolytic cleavage of the ectodomain of its glycosyl phosphatidylinosital-anchored counterpart that is situated on the luminal cell surface of the loop of Henle. Plasmatic UMOD levels correlate with kidney function (Steubl et al., 2016). UMOD has been found in uEVs (Pisitkun et al., 2004).

## 6 Metabolism-related proteins

Metabolism-related proteins play a crucial role in the development of CKD, with particular significance in OCKD. Dysregulation of these proteins, involved in nutrient processing and energy balance, can lead to metabolic abnormalities, oxidative stress, and inflammation in the kidneys, exacerbating CKD progression in obese individuals. In this section, we listed metabolism proteins that have been related to OCKD.

SGLT2. Solute carrier family 5 member 2 (SGLT2) is a electrogenic Na (+)-coupled sugar simporter that actively transports D-glucose at the plasma membrane, with a Na (+) to sugar coupling ratio of 1:1. Hyperglycaemia is implicated in the development of glomerular hypertension and hyperfiltration by enhancing sodium reabsorption in the proximal tubule through SGLT2 (Docherty et al., 2020).

FABP3. Fatty acid binding protein 3 (FABP3) plays a role in the intracellular transport of long-chain fatty acids and their acyl-CoA esters. Circulating levels of FABP3 has been related to CKD in diabetic patients (Yu et al., 2022). In urine, FABP3 levels are related to acute kidney injury (Dihazi et al., 2016).

TRPC6. Transient receptor potential cation channel subfamily C member 6 (TRPC6) is a receptor-activated non-selective calcium permeant cation channel. Mutations and over-activation in TRPC6 channel activity lead to the development of glomeruli injury. TRPC6 activity is related to de development of diabetes kidney disease (Staruschenko et al., 2019). TRPC6 levels in uEVs have been proposed as potential biomarkers of glomerular disease (Hogan et al., 2014).

LRP2. LDL receptor related protein 2 (LRP2, also known as megalin) is a multi-ligand endocytic receptor that is expressed in many different tissues but primarily in absorptive epithilial tissues such as the kidney. Megalin-mediated tubuloglomerular alterations in high-fat diet-induced kidney disease (Kuwahara et al., 2016). Moreover, PPARα/γ and their agonists positively control megalin expression in the kidneys (Cabezas et al., 2011). Megalin has been found in uEVs (Pisitkun et al., 2004).

CUBN. Cubilin (CUBN) is an endocytic receptor which plays a role in lipoprotein, vitamin, and iron metabolism by facilitating their uptake. In high fat diet, CUBN mediates the ectopic fat accumulation in the kidney (Lubojemska et al., 2021). CUBN is present in uEVs (Hogan et al., 2014).

GLUT1. Solute carrier family 2 member 1 (GLUT1) is a facilitative glucose transporter responsible for constitutive or basal glucose uptake. Alteration in the cellular trafficking of GLUT1 in kidney cells are related to the development of diabetic kidney disease (Gnudi and Raij, 2006; Wasik and Lehtonen, 2018).

GLUT2. Solute carrier family 2 member 2 (GLUT2) is a facilitative hexose transporter that mediates the transport of glucose, fructose, and galactose. Recently, GLUT2 has emerged as a central regulator in the pathogenesis of diabetic kidney disease (Ahmad et al., 2022).

GLUT4. Solute carrier family 2 member 4 (SLC2A4) is an insulin-regulated facilitative glucose transporter, which plays a key role in removal of glucose from circulation. Altered kidney levels of GLUT4 have been found in patients with diabetic kidney disease (Piwkowska et al., 2022). We previously demonstrated that EVs derived from cardiomyocytes have GLUT4 on their surface with functional consequences for the cardio-endothelial communication axis (Garcia et al., 2016).

TM6SF2. Transmembrane 6 superfamily member 2 (*TM6SF2*) encodes a protein of undetermined function. Children with obesity carrying the TM6SF2 167K allele show higher eGFR levels compared with E167 allele homozygous subjects, independently of non-alcoholic fatty liver disease (NAFLD) (Marzuillo et al., 2020; Liu et al., 2023).

CNR1. Cannabinoid receptor 1 (CNR1) is a G-protein coupled receptor for endogenous cannabinoids (eCBs), including N-arachidonoylethanolamide and 2-arachidonoylglycerol (2-AG), as well as phytocannabinoids, such as delta (Polidori et al., 2020)-tetrahydrocannabinol (THC). The activity of CNR1 has been repeatedly shown to contribute to both diabetic and non-diabetic CKD. Interestingly, recent reports of acute kidney injury (AKI) have been attributed to synthetic cannabinoid use (Arceri et al., 2023).

## 7 Proteins related to inter-tissue crosstalk

Inter-tissue crosstalk mechanisms play a pivotal role in the pathogenesis of CKD, with particular emphasis on OCKD. Evidence from various studies highlights the intricate interplay between adipose tissue, kidneys, and other organs involved in the progression of OCKD. These mechanisms involve the secretion of pro-inflammatory cytokines, adipokines, EVs and other mediators that promote renal inflammation, fibrosis, insulin resistance, and kidney lipid accumulation. In this section we listed EV surface proteins potentially related to inter-tissue crosstalk in the development of OCKD.

ADIPOQ. Adiponectin (ADIPOQ) is an adipokine involved in the control of fat metabolism and insulin sensitivity, with direct anti-diabetic, anti-atherogenic and anti-inflammatory activities. Adiponectin is closely related to the development of OCKD (Przybycinski et al., 2020). Serum levels of adiponectin could be used as biomarker of renal dysfunction (Song et al., 2020). Preliminary evidence from flow cytometric analyses showed that cEVs contain the adipocyte marker adiponectin. This implies that adiponectin was detected on the surface of cEVs (Phoonsawat et al., 2014; Gustafson et al., 2015).

HSD11B2. Hydroxysteroid 11-beta dehydrogenase 2 (HSD11B2) catalyzes the conversion of biologically active 11beta-hydroxyglucocorticoids (11beta-hydroxysteroid) such as cortisol, to inactive 11-ketoglucocorticoids (11-oxosteroid) such as cortisone, in the presence of NAD (+).11βHSD1 amplifies glucocorticoid action in cells and contributes to hypertension through direct and indirect effects on the kidney and vasculature in a metabolic syndrome context (Bailey, 2017).

CD36 is a multifunctional glycoprotein that acts as receptor/transporter for a broad range of ligands. Renal CD36 is mainly expressed in tubular epithelial cells, podocytes and mesangial cells, and is markedly upregulated in the setting of CKD and OCKD, contributing to kidney fat accumulation (Yang et al., 2017). We showed that CD36 is expressed on the surface of cEVs and it is related with the delivery of free fatty acids (FFA) from blood flow to the heart (Garcia et al., 2019). Bariatric surgery resulted in significantly altered levels of CD36 in cEVs of monocyte and endothelial origin (Botha et al., 2018).

PTHrP and PTH1R. (Parathyroid hormone-related protein and Parathyroid hormone receptor). Parathyroid hormone is a neuroendocrine peptide which is a critical regulator of cellular and organ growth, development, migration, differentiation, and survival and of epithelial calcium ion transport. PTH1R is a member of the G-protein coupled receptor family 2. This protein is a receptor for parathyroid hormone and for parathyroid hormone-related peptide. PTHrP and PTH1R modulation in adipose tissue mediates cachexia (wasting syndrome associated with elevated basal energy expenditure and loss of adipose and muscle tissues) in models of kidney failure (Ki et al., 2016). EVs from Lewis lung carcinoma cells induces lipolysis and adipose tissue browning in cachecia via PTHrP/PTH1R (Hu et al., 2021).

PHGDH. Phosphoglycerate dehydrogenase (PHGDH) catalyzes the reversible oxidation of 3-phospho-D-glycerate to 3-phosphonooxypyruvate, the first step of the phosphorylated L-serine biosynthesis pathway. Phosphoglycerate dehydrogenase and serine levels are markedly downregulated in human subjects with diabetic kidney disease or obesity-related renal dysfunction. Oral administration of serine ameliorates high-fat diet induced fatty liver and renal dysfunction, suggesting a potential approach against obesity related metabolic disorders (Chen et al., 2022).

PLIN2. Perilipin 2 (PLIN2) is a structural component of lipid droplets, which is required for the formation and maintenance of lipid storage droplets. This protein is associated with the lipid globule surface membrane material, and maybe involved in development and maintenance of adipose tissue. Perilipin 2 impacts acute kidney injury via regulation of PPARα (Xu et al., 2021). Interestingly, perilipin A in cEVs derived from adipose tissue has been proposed as biomarkers for adipose tissue health (Eguchi et al., 2016).

## 8 Proteins related to kidney fibrosis

Kidney fibrosis is a key process in the progression CKD, particularly in cases related to obesity (OCKD). Excessive adipose tissue in obesity triggers a cascade of inflammatory responses and oxidative stress, leading to renal damage. In OCKD, persistent renal inflammation triggers excessive deposition of extracellular matrix proteins, leading to fibrosis. This pathological process disrupts normal kidney function, impairs filtration, and compromises nephron integrity, ultimately contributing to the decline of renal function. In this section we asses EV surfaces proteins related to fibrosis development in CKD and OCKD.

PROM1. Promilin 1 (PROM1) is a pentaspan transmembrane glycoprotein that binds cholesterol in cholesterol-containing plasma membrane microdomains and may play a role in the organization of the apical plasma membrane in epithelial cells. PROM1 has been proposed as a biomarker of kidney fibrosis in different kidney pathologies (Hu et al., 2022). Moreover, acute and chronic glomerular damage associates with reduced PROM1 expression in uEVs (Dimuccio et al., 2020).

KL (Klotho) is a membrane-bound protein predominantly expressed in the kidney, where it acts as a permissive co-receptor for Fibroblast Growth Factor 23. In its shed form, Klotho exerts anti-fibrotic effects in several tissues. Klotho overexpression or supplementation protects against fibrosis in various models of renal fibrotic disease (Zou et al., 2018). uEVs carrying Klotho improve renal function in an acute tubular injury model (Grange et al., 2020).

The NOTCH pathway, known for its evolutionary conservation, serves as a crucial ligand-receptor signaling mechanism implicated in the control of tissue homeostasis, the maintenance of adult stem cells, and the normal development of vasculature as well as angiogenesis (Zhou et al., 2022). Activation of Notch signaling occurs through the interaction of specific transmembrane Notch ligands, which can be of the Jagged or Delta-like type, located on adjacent cells, engaging with the extracellular domain of the receptor when they are in close proximity. In mammals, four Notch receptors have been identified, namely, Notch1-4, and they are triggered by five well-documented canonical ligands, including Delta-like 1, 3, and 4 (Dll1, Dll3, and Dll4) as well as Jagged1 and Jagged2 (Sassoli et al., 2011; Olsauskas-Kuprys et al., 2013). Numerous investigations have underscored the pivotal role played by the Notch signaling pathway in the regulation of chronic kidney disease (CKD) progression, particularly in the context of fibrotic processes (Han et al., 2017). It is worth noting that all components of the NOTCH pathway, both the ligands and the receptors, have been functionally detected in extracellular vesicles (EVs) from diverse sources (Gonzalez-King et al., 2017; Gonzalez-King et al., 2022).

TGFB1. Transforming growth factor beta 1 (TGFB1) a secreted ligand of the TGF-beta (transforming growth factor-beta) superfamily of proteins. Ligands of this family bind various TGF-beta receptors leading to recruitment and activation of SMAD family transcription factors that regulate gene expression. TGFB1 regulation is directly linked to liver fibrosis. Growing evidence supports protective effects of TGF-β by mechanisms which include inhibiting inflammation and induction of autophagy (Sureshbabu et al., 2016). TGFB1 is not a transmembrane protein but was included due to evidence supporting the presence of functional TGFB1 on the surface of EVs (Shelke et al., 2019).

WNT5A. Wnt family member 5A (WNT5A) is a ligand for members of the frizzled family of seven transmembrane receptors. Can activate or inhibit canonical Wnt signaling, depending on receptor context. WNT5A activity is related to tubular inflammation in diabetic nephropathy (Li et al., 2021a) and kidney fibrosis (Feng et al., 2018). WNT5A has been found on the surface of EVs and related to pulmonary fibrosis (Martin-Medina et al., 2018).

CTNNB1. Catenin beta 1 (CTNNB1) is a key downstream component of the canonical Wnt signaling pathway. Wnt/β-catenin signaling is a master driver for in renal fibrogenesis, and recent works has proposed blocking this signaling may benefit renal interstitial fibrosis (Li et al., 2021b).

CXCR4. C-X-C motif chemokine receptor 4 (CXCR4) is a G-protein coupled receptor that transduces a signal by increasing intracellular calcium ion levels and enhancing MAPK1/MAPK3 activation. CXCR4 has been directly related to kidney fibrosis via multiple effectors (Yuan et al., 2015). Horizontal transference of CXCR4 by EVs promote hepatocarcinoma cell migration, invasion, and lymphangiogenesis (Li et al., 2018).

## 9 Proteins related to kidney inflammation and immunity

Inflammation is a hallmark of CKD, initiating and propagating renal injury. Immune cells infiltrate the renal microenvironment, releasing pro-inflammatory cytokines and triggering oxidative stress. This cascade results in endothelial dysfunction, fibrosis, and disrupted glomerular filtration (Mihai et al., 2018). Notably, immune dysregulation plays a pivotal role, as both innate and adaptive immune responses contribute to CKD pathogenesis. Lymphocytes, macrophages, and dendritic cells orchestrate a complex interplay, amplifying tissue damage and fostering a maladaptive repair response. OCKD exemplifies the intricate relationship between metabolic dysfunction and renal deterioration. Adipose tissue-derived adipokines fuel systemic inflammation, promoting insulin resistance and cytokine release (Espi et al., 2020). This inflammatory milieu spurs renal inflammation and fibrosis, exacerbating nephron damage. Moreover, immune cells within adipose depots contribute to systemic inflammation, bridging the connection between obesity and CKD (Wang et al., 2022a). In this section we have listed EV surface proteins related to CKD and OCKD that could potentially serve as biomarkers.

TLR2. Toll-like receptor 2 (TLR2). Toll-like receptor (TLR) family plays a fundamental role in pathogen recognition and activation of innate immunity. Speer et al., showed that abnormal high-density lipoprotein induces endothelial dysfunction via activation of Toll-like receptor-2 in CKD patients Speer et al. (2013).

CR1. Complement C3b/C4b receptor 1 (CR1) is a membrane immune adherence receptor that plays a critical role in the capture and clearance of complement-opsonized pathogens by erythrocytes and monocytes/macrophages. Increased evidence suggested that the complement system, the most important and fundamental component of innate immune responses, is actively involved in the development of metabolic kidney diseases, in particular OCKD (Xu et al., 2022). CR1 has been found in uEVs (Prunotto et al., 2013).

WNT1. Wnt family member 1 (WNT1) is a ligand for members of the frizzled family of seven transmembrane receptors. Acts in the canonical Wnt signaling pathway by promoting beta-catenin-dependent transcriptional activation. WNT1-inducible signaling pathway protein 1 regulates kidney inflammation through the NF-κB pathway (Wang et al., 2022b). Eventhough WNT1 is not a transmembrane protein, it was included because increased evidence is showing EVs as functional carriers of different ligands and receptors of the WNT/β-catenin pathway (Gross and Zelarayan, 2018).

CXCR7. Atypical chemokine receptor 3 (CXCR7) controls chemokine levels and localization via high-affinity chemokine binding that is uncoupled from classic ligand-driven signal transduction cascades, resulting instead in chemokine sequestration, degradation, or transcytosis. Also known as interceptor (internalizing receptor) or chemokine-scavenging receptor or chemokine decoy receptor. CXCR7 regulates CXCR4 expression and capillary tuft development in kidney (Haege et al., 2012).

CXCL10. C-X-C motif chemokine ligand 10 (CXCL10) is a pro-inflammatory cytokine that is involved in a wide variety of processes such as chemotaxis, differentiation, and activation of peripheral immune cells, regulation of cell growth, apoptosis, and modulation of angiostatic effects. Cxcl10 deficiency attenuates renal interstitial fibrosis through regulating epithelial-to-mesenchymal transition (Gao et al., 2022). Interestingly, by its role in inflammation, CXCL10 has been proposed as a potential biomarker and therapeutic target in human kidney disease (Gao et al., 2020). In diabetes, pancreatic beta cells in a pro-inflammatory environment release EVs with CXCL10 on the surface, which induce failure of neighboring beta cells through activation of the CXCL10/CXCR3 axis (Javeed et al., 2021).

## 10 Perspective

EVs have surfaced as promising indicators for CKD and OCKD, given their active participation in the etiology and progression of these conditions. Consequently, EVs have been implicated in a range of processes linked to kidney pathophysiology, including metabolic alterations, inflammatory responses, and fibrotic transformations. The distinctive assortment of surface proteins on EVs renders them alluring candidates in the realm of biomarker discovery. One particularly advantageous aspect of EVs as biomarkers lies in their presence in both urine (uEVs) and circulation (cEVs), providing a minimally invasive avenue for acquiring kidney disease-related insights. However, the identification and analysis of EV specimens pose considerable challenges, primarily due to their heterogeneity and low abundance. Thus, specific surface proteins on EVs associated with kidney disorders could bolster their diagnostic potential. In this context, uEVs and cEVs exhibit a cell-specific origin, thereby reflecting their distinctive expression of EV surface proteins. This facet opens up the prospect of investigating specific EV subgroups, yielding more precise results. For instance, our work demonstrated substantial alterations in CD36 levels in cEVs originating from monocyte and endothelial cells following bariatric surgery (Botha et al., 2018).

Conventional methodologies for assessing EV proteins, such as Western Blot, mass spectrometry, or ELISA, exhibit limitations in terms of throughput and specificity. However, emerging technologies like EV-Array, Exoview, or HS-FCM offer the capacity for high-throughput analysis and multiplex EV detection, permitting simultaneous assessment of multiple surface proteins. These technologies hold the potential to facilitate the integration of EV-based biomarkers into clinical practice. Furthermore, the development of innovative technologies, including microfluidics and nanomaterials, holds promise in enhancing the sensitivity and specificity of EV detection.

EV based biomarkers hold considerable potential for improving the diagnosis and monitoring of kidney pathology. The application of these EV protein biomarkers in clinical practice has the ability to enhance early detection, facilitate timely interventions, and improve patient outcomes. Combining multiple biomarkers and clinical parameters may provide a more accurate picture of kidney health. However, further validation studies are needed to confirm the diagnostic and prognostic value of EV surface proteins in larger patient cohorts.
